# Effect of Kinematic Analysis on the Gait of School-Going Children With Different Types of Foot Arches: An Observational Study Using Xsens 3D Motion Technology

**DOI:** 10.7759/cureus.78322

**Published:** 2025-02-01

**Authors:** Siddhi G Rathi, H V Sharath, Pradhyum D Kolhe

**Affiliations:** 1 Department of Paediatric Physiotherapy, Ravi Nair Physiotherapy College, Datta Meghe Institute of Higher Education and Research (Deemed to be University), Wardha, IND

**Keywords:** flat foot, gait, high arch, normal arch, pediatric pes planus, pes planus, posture

## Abstract

Background

Gait mechanics can be influenced by foot structure, particularly in children with different types of arches, such as normal arches, flat feet, and high arches. Understanding how foot structure affects gait is essential for developing targeted interventions to improve walking efficiency and reduce injury risk. This observational study uses Xsens 3D motion technology (Xsens Technologies B.V., Netherlands) to analyze the impact of different foot arches on gait in school-going children.

Objective

The aim of the study is to investigate the effect of foot arch types on gait variables, such as speed, step length, cadence, and gait phases, in school-going children, using advanced kinematic analysis.

Methods

A total of 558 children were classified into three groups based on their foot arch type: normal arch, flat foot, and high arch. Kinematic data was collected using Xsens 3D motion technology to analyze gait variables. One-way ANOVA was used to compare continuous variables (age, height, weight, body mass index (BMI)) between the groups, while the chi-square test was used for categorical variables (gender). Gait variables were analyzed using one-way ANOVA and Tukey’s post-hoc test.

Results

No significant differences were observed between the groups in terms of age, height, weight, BMI, or gender (p > 0.05). However, significant differences were found in all gait variables (p < 0.0001). Flat-footed individuals exhibited the highest walking speed (1.03 m/s), step count (34.01 steps/min), and step length (7.50 m), while high-arch participants demonstrated slower speeds (0.96 m/s) and wider step width (-0.16 m). High-arch individuals also spent more time in the double and single support phases, indicating a need for increased balance during gait.

Conclusion

Foot arch type significantly affects gait mechanics in school-going children. Children with flat feet exhibit faster gait speeds and longer step lengths, while those with high arches demonstrate slower, more cautious walking patterns. These results highlight the importance of individualized interventions, such as orthotics or gait training, to optimize walking efficiency and reduce the risk of gait-related issues in children with abnormal foot arches.

## Introduction

The foot, renowned as a sophisticated 'terminal organ,' boasts remarkable weight-bearing and locomotion capabilities. Comprising 26 bones and 33 joints, intricately supported by muscles, tendons, and ligaments, it can be categorized into hindfoot, midfoot, and forefoot. The foot's two longitudinal arches, shaped by the cuboid, navicular, and three cuneiform bones, enhance walking and running efficiency through their elastic properties, minimizing energy consumption and mitigating the impact on the proximal lower limb [1-5]. Pes cavus, or pes planus, are examples of unbalanced, functionally unstable foot disorders caused by deviations from the medial longitudinal arch, the elevated arch attribute of pes cavus, and the flat arch attribute of pes planus [2]. When the medial longitudinal arch collapses, the forefoot abducts, and the hindfoot everts, resulting in a flat foot. Deviations in lower extremity kinematics distinguish it during dynamic activity [6]. Flat feet can be classified into two main categories: flexible and rigid flat feet. Usually appearing in childhood, flexible flat feet can persist into adulthood. When a flat foot is said to be "flexible," it indicates that when weight is removed, the foot arch returns to its usual position [7].

The general population is estimated to have a 25% prevalence of flat foot based on prior research [8]. Flat feet are more common in women, those with body mass indices (BMIs), and people with bigger feet. The prevalence of flat foot drastically decreases with age: it is 54% at three years old, 24% at six years old, and 11.25% at eighteen to twenty-five years old [8]. The underlying etiology of pediatric flexible flat foot has not been found. Regarding its etiology, two conventional theories have been described. Reduced foot muscle strength is one idea that explains flexible flat feet. Another idea states that the strength and structure of the osseous-ligament complex primarily form the arch. The discovery that the lack of a normal medial arch during weight-bearing is commonly associated with the incompetence of the spring ligament lends credence to the latter theory [9,10].

An orthopedic ailment known as pes cavus can affect both adults and children. Since the cavovarus presentation is the most typical way that the cavus foot manifests itself, pes cavus and pes cavovarus are frequently used interchangeably. Forefoot pronation, plantar flexion of the first ray, elevation of the medial longitudinal arch, valgus, hindfoot varus, and forefoot adduction are common characteristics of pes cavus deformity. A hindfoot deformity, a forefoot-driven deformity (a flexed first ray), or a combination of both pathologies can cause a cavovarus foot [11-13].

Gait changes and postural variations accompany foot deformity, with advancements in technology offering tools like the Xsens motion vector notation (MVN) gait report (Xsens Technologies B.V., Netherlands) for cost-effective and cloud-based gait analysis. This report delves into spatial and temporal gait parameters, angular kinematics, and joint kinetics. The increased prevalence of foot deformity due to various causative factors contributes significantly to these gait changes in affected individuals.

## Materials and methods

An observational comparison study was carried out at the Centre for Advanced Physiotherapy Education and Research (CAPER) at Ravi Nair Physiotherapy College in Wardha, Maharashtra, India. The interested participants were informed of the study's purpose and gave their consent. Around 186 children with flat feet, high arches, and normal arches were involved in the study. Based on the inclusion and exclusion criteria (Table 1), participants were added to the survey. The patient gave informed consent, and each participant's demographic data was collected. The Indian Ink Print Method, or the Navicular Drop Test, has been used to determine the foot arch type. Every group's gait pattern has been monitored using the Xsens MVN system. Seventeen distinct locations for sensors were used on the body. One each at the head, sternum, and lumbar area. Two sensors were placed at the posterior portion of the scapular region, elbow, forearm, and wrist of the upper limb. Two sensors were placed at the ankle, knee, and hip joints of the lower limb. Participants were asked to walk for a distance of 20 to 30 meters. The gait's temporal and spatial properties were noted.

**Table 1:** Inclusion and exclusion criteria

Inclusion criteria	Exclusion criteria
Age between 7 to 15 years	Any neurological conditions
Both male and female	Psychiatric illness
No recent injuries and recent trauma	Limb length discrepancy
No history of any surgeries in their lifetime	Plantar fasciitis and any musculoskeletal injuries

Sample size

The formula below is used for determining the sample size,

$n= \frac{Z^2_{1-\alpha/2} \cdot p(1-p)}{d^2}$

Where,

Alpha (α) = 0.05

p = prevalence of abnormal foot arch = 14% i.e., estimated proportion (p) = 0.14

d = denied error of margin= 5%= 0.05

N = 186 patients needed

Statistical analysis

The study employed both descriptive and inferential statistics, utilizing the chi-square test, one-way ANOVA, and multiple comparisons: Tukey test. The software used for the analysis included IBM SPSS Statistics for Windows, Version 27 (Released 2020; IBM Corp., Armonk, New York, United States) and GraphPad Prism 7.0 versions (Insight Venture Management, LLC, New York, US), with a significance threshold of p < 0.05.

Ethical considerations

The Datta Meghe Institute of Higher Education and Research’s Institutional Ethics Committee authorized this study on December 20, 2023 (Ref no: DMIHER(DU)/IEC/2023/100). The confidentiality of the participants in this study was preserved, and all procedures followed ethical standards for research.

## Results

For continuous variables (age, height, weight, BMI), ANOVA was used to compare means between the groups (indicated by F-values). All p-values are greater than 0.05, indicating no statistically significant differences between the groups for these variables. For the categorical variable (gender), the chi-square test (Χ²-value) was used. Similarly, p = 0.32 shows no statistically significant difference between the gender distribution across foot types (Table 2).

**Table 2:** Distribution of patients according to the demographic characteristics in three groups (one-way ANOVA) S: significant; NS: not significant; BMI: body mass index

	Normal Arch	Flat Foot	High Arch	F-value
Age (yrs)	10.89±2.49	10.92±2.43	10.98±2.46	0.05,p=0.94,NS
Height (cm)	144.06±14.53	143.77±13.68	143.99±13.95	0.02,p=0.97,NS
Weight (kg)	37.24±11.16	36.94±10.48	37.10±10.62	0.03,p=0.96,NS
BMI (kg/m^2)^	17.42±2	17.41±1.80	17.45±1.82	0.01,p=0.98,NS
Gender				Χ2-value
Male	96(51.61%)	82(44.09%)	86(46.24%)	2.24,p=0.32,NS
Female	90(48.39%)	104(55.91%)	100(53.76%)	

The analysis of gait variables across foot types, normal arch, flat foot, and high arch reveals significant differences in walking mechanics, as indicated by p-values of 0.0001 for all variables. Individuals with flat feet have the highest walking speed, averaging 1.03 m/s, while those with high arches exhibit the lowest speed at 0.96 m/s. Similarly, flat-foot participants have the highest step count (34.01 steps/min) and longest step length (7.50 m), compared to 31.52 steps/min and 7.35 m for high-arch individuals. These results suggest that flat-footed individuals may adjust their gait by increasing their speed and step length to compensate for structural differences.

Differences are also notable in gait phases. Flat-foot individuals spend more time in the swing phase (0.043) and less time in the stance phase (-0.022), while high-arch individuals spend more time in both the double support (-0.041) and single support phases (-0.037), compared to normal arch and flat-foot participants. This implies that high-arch individuals may require additional support time for balance. Furthermore, step width is widest in high-arch individuals (-0.16 m) and narrowest in flat-foot participants (-0.09 m), indicating lateral adjustments during walking based on foot structure.

Overall, the significant differences in gait variables such as speed, cadence, and step length highlight the influence of foot types on walking patterns. These findings point to compensatory strategies employed by those with flat feet and high arches to maintain an efficient gait. Understanding these patterns is essential for clinicians when recommending interventions such as gait training, orthotics, or specialized footwear to improve walking mechanics and reduce the risk of gait-related issues (Table 3).

**Table 3:** Comparison of gait variables in three groups (one-way ANOVA and multiple comparison Tukey test)

Gait Variables	Normal Arch	Flat Foot	High Arch	F-value	P-value
Speed (m/s)	0.97±0.007	1.03±0.05	0.96±0.007	218.03	0.0001,S
Steps (per minute)	32.71±0.99	34.01±0.72	31.52±0.63	449.75	0.0001,S
Step Length (m)	7.41±0.01	7.50±0.009	7.35±0.01	5338.07	0.0001,S
Stride Length (m)	0.017±0.005	0.034±0.007	0.012±0.002	957.94	0.0001,S
Step Width (m)	-0.12±0.04	-0.09±0.01	-0.16±0.04	166.23	0.0001,S
Swing Phase	0.029±0.005	0.043±0.004	0.015±0.005	1342.41	0.0001,S
Stance Phase	-0.027±0.006	-0.022±0.004	-0.035±0.005	284.47	0.0001,S
Cadence (steps/min)	115.52±0.32	112.02±1.08	108.57±1.24	2381.09	0.0001,S
Double Support Phase	-0.038±0.005	-0.035±0.005	-0.041±0.007	39.71	0.0001,S
Single Support Phase	-0.027±0.005	-0.022±0.004	-0.037±0.004	479.95	0.0001,S

## Discussion

The present study aimed to evaluate the effect of foot arch types, normal arch, flat foot, and high arch on gait parameters in school-going children, using Xsens 3D motion technology for kinematic analysis. The findings reveal significant differences in gait mechanics between the foot types, providing insights into compensatory strategies employed by children with flat feet and high arches to maintain efficient walking patterns. The analysis of demographic characteristics (age, height, weight, BMI) using one-way ANOVA demonstrated no statistically significant differences between the three groups, as all p-values were greater than 0.05. This suggests that the differences observed in gait variables are likely related to the structural differences in foot arches rather than being influenced by demographic factors such as age, height, or body composition [14-18].

Similarly, the chi-square test for gender distribution (p = 0.32) indicated no significant gender difference across the foot types, reinforcing the notion that the observed gait variations are primarily related to the foot arch structure rather than gender differences. The kinematic analysis revealed significant differences in gait variables such as speed, cadence, step length, stride length, and phases of gait across the three-foot types. Individuals with flat feet demonstrated the highest walking speed (1.03 m/s), cadence (34.01 steps/min), and step length (7.50 m), which suggests that flat-footed children may increase their walking speed and lengthen their steps to compensate for the lack of arch support. This finding aligns with previous research indicating that flat-footed individuals often adjust their gait mechanics to maintain stability and walking efficiency [19,20].

In contrast, children with high arches had the lowest walking speed (0.96 m/s) and cadence (31.52 steps/min), suggesting a more cautious gait pattern. High-arch individuals also exhibited the widest step width (-0.16 m), which may indicate that they compensate for structural rigidity by widening their stance to improve balance and stability during walking. The longer time spent in double support (-0.041) and single support (-0.037) phases further suggests that high-arch individuals rely more on support time to ensure balance, as compared to their flat-footed counterparts. The significant differences in swing and stance phases across foot types highlight how structural variations in the foot impact the gait cycle. Flat-footed children spent more time in the swing phase (0.043) and less time in the stance phase (-0.022), which may indicate a more dynamic and rapid gait, possibly compensating for the decreased foot rigidity. Conversely, high-arch individuals showed longer durations in the stance and support phases, possibly due to the increased need for stability and balance. This may be attributed to the rigid nature of a high arch, which could limit flexibility during gait transitions.

Understanding these compensatory strategies is essential for clinicians and therapists. Children with flat feet may benefit from interventions such as orthotics, which can provide additional arch support, improve stability, and optimize walking mechanics. Similarly, those with high arches could benefit from gait training exercises aimed at improving balance and reducing the strain associated with prolonged support phases. The kinematic differences observed in this study also underline the importance of personalized interventions for children with abnormal foot arches. Flat-footed individuals may require strategies that focus on reducing the increased stress caused by higher step counts and stride lengths, while high-arch individuals may need support to mitigate the rigidity and limited flexibility of their feet.

Study limitations

While the use of Xsens 3D motion technology provided a comprehensive analysis of gait patterns, the study had some limitations. The sample size, though substantial, was restricted to a specific age group of school-going children, limiting the generalizability of the findings to other populations. Additionally, other factors such as muscle strength, joint flexibility, and physical activity levels were not controlled for in this study, and these could influence gait mechanics. Future research may consider including these variables to gain a more holistic understanding of how foot structure affects gait in children.

## Conclusions

This observational study demonstrates that foot arch structure significantly affects gait mechanics in school-going children. Children with flat feet adopt compensatory strategies such as increasing walking speed and step length, while those with high arches exhibit a more cautious and balanced gait. The use of Xsens 3D motion technology allowed for precise kinematic analysis, revealing key insights into how different foot types influence walking patterns. These findings underscore the need for individualized gait interventions to optimize walking efficiency and prevent potential gait-related issues in children with abnormal foot arches.
